# Molecular subtyping of Alzheimer’s disease with consensus non-negative matrix factorization

**DOI:** 10.1371/journal.pone.0250278

**Published:** 2021-05-20

**Authors:** Chunlei Zheng, Rong Xu

**Affiliations:** 1 Center for Artificial Intelligence in Drug Discovery, School of medicine, Case Western Reserve University, Cleveland, Ohio, United States of America; 2 Case Comprehensive Cancer Center, Case Western Reserve University, Cleveland, Ohio, United States of America; McGill University, CANADA

## Abstract

Alzheimer’s disease (AD) is a heterogeneous disease and exhibits diverse clinical presentations and disease progression. Some pathological and anatomical subtypes have been proposed. However, these subtypes provide a limited mechanistic understanding for AD. Leveraging gene expression data of 222 AD patients from The Religious Orders Study and Memory and Aging Project (ROSMAP) Study, we identified two AD molecular subtypes (synaptic type and inflammatory type) using consensus non-negative matrix factorization (NMF). Synaptic type is characterized by disrupted synaptic vesicle priming and recycling and synaptic plasticity. Inflammatory type is characterized by disrupted IL2, interferon alpha and gamma pathways. The two AD molecular subtypes were validated using independent data from Gene Expression Omnibus. We further demonstrated that the two molecular subtypes are associated with APOE genotypes, with synaptic type more prevalent in AD patients with E3E4 genotype and inflammatory type more prevalent in AD patients with E3E3 genotype (p = 0.031). In addition, two molecular subtypes are differentially represented in male and female AD, with synaptic type more prevalent in male and inflammatory type in female patients (p = 0.051). Identification of AD molecular subtypes has potential in facilitating disease mechanism understanding, clinical trial design, drug discovery, and precision medicine for AD.

## Introduction

Alzheimer’s disease (AD) is the most common neurodegenerative disease in elderly population, characterized by pathological extracellular deposition of beta-amyloid (Aβ) peptides and intracellular tau protein fibers in the brain [1]. AD is a heterogenous and multifactorial disease, with diverse clinical presentations in different affected brain areas (left and right cerebral hemispheres as well as anterior-posterior axis) [2–5], different phenotypes (dysexecutive, amnesic and aphasic) [6, 7], and different rates of disease progression [8]. Recent studies suggested that Aβ aggregates in different biochemical composition [9]. Defining subtypes of AD is important for disease mechanism understanding, clinical trial design, drug discovery, and personalized treatments.

Neuroimaging, beta amyloid and tau have been used for AD subtyping [9–13], however, subtypes identified based on image analysis and beta amyloid offer limited mechanistic understanding into AD pathophysiology. High-throughput genomic data has greatly expanded our understanding for disease mechanism of AD. Genome-wide association studies (GWAS) have initially identified over 20 loci for late-onset AD [14, 15]. A recent approach called genome-wide association-by-proxy (GWAX) using larger sample size has further expanded the susceptibility loci of AD to 40 [16–18]. Several pathways or molecular networks involved in AD were identified using gene expression data [19, 20]. In addition, advanced machine learning and statistical methods have used genomic data to classify AD from normal and mild cognitive impairment (MCI) or predicting MCI to AD conversion [21–24]. However, genomic data have not been used for AD subtyping.

The Religious Orders Study and Memory and Aging Project (ROSMAP) is a longitudinal clinical-pathologic cohort study of aging and AD [25]. Currently, around 2,500 individuals were involved in this study and genomic data from 642 participants are available. In this study, we leveraged these valuable data for AD molecular subtyping using non-negative matrix factorization (NMF) clustering method. It has been shown that NMF-based classification is accurate and robust for clustering of genomic data as compared to other methods [26]. NMF has been used in cancer molecular subtyping [27, 28]. In this study, we applied NMF to identify molecular subtypes of AD using gene expression data from ROSMAP and validated the AD molecular subtype in independent datasets. We also investigated the association of AD molecular subtype with patient demographic, clinical and APOE status variables.

## Materials and methods

**Fig 1:**
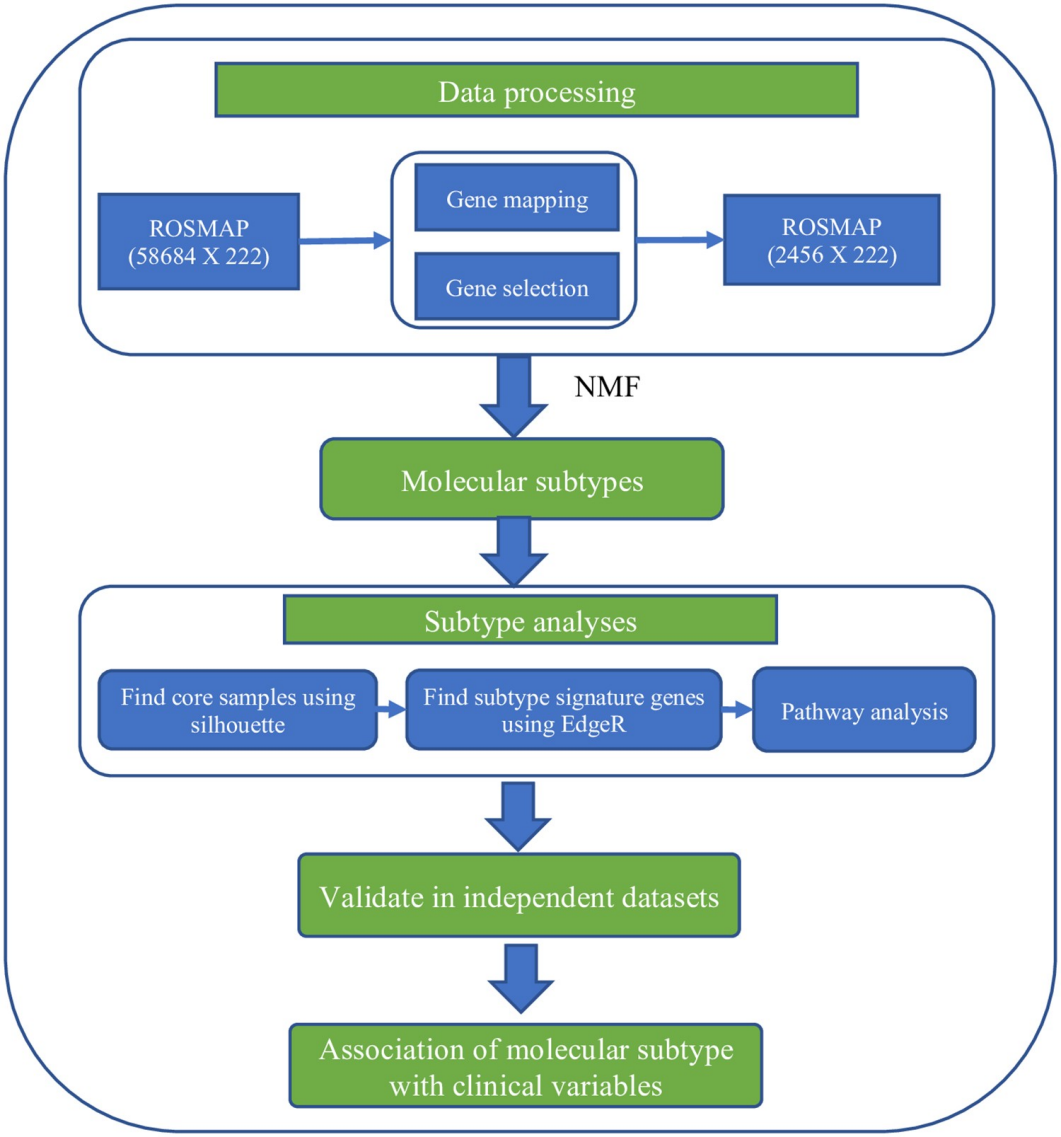
Overview of the methods. NMF: non-negative matrix factorization.

The overall methods were illustrated in Fig 1. The Religious Orders Study and Memory and Aging Project (ROSMAP) was used as the discovery dataset. First, we applied consensus matrix-based NMF into ROSMAP to identify AD molecular subtypes. Second, subtype analysis was performed to identify signature genes and enriched pathways for each molecular subtype. Third, we validated these molecular subtypes in independent datasets (GEO). Finally, we investigated the association of AD molecular subtype with available demographic and clinical variables, and APOE genotype.

### ROSMAP data

ROSMAP contains 222 participants with clinical consensus diagnosis of AD at time of death. Raw gene expression data from frontal cortex and corresponding clinical data were downloaded from synapse.org (syn3219045). Raw count data were normalized and processed according to commonly used procedure described in edgeR (version: 3.28.0) [29, 30]. Data were first normalized by sequencing library size. Non-expressed genes, defined as count per million less than 5 in 80% of samples, were then filtered out, resulting in 12281 genes. To obtain a robust classifier and also reduce the number of genes for NMF-based clustering, we experimented with the different cutoffs ranging from top 10% to 40% (1228 to 4912 genes) based on their interquartile range (IQR) for clustering. While the obtained results were very similar, we presented the clustering result using the top 20% cutoff (2456 genes).

### Consensus NMF for AD molecular subtyping

#### Non-negative matrix factorization

**Fig 2:**
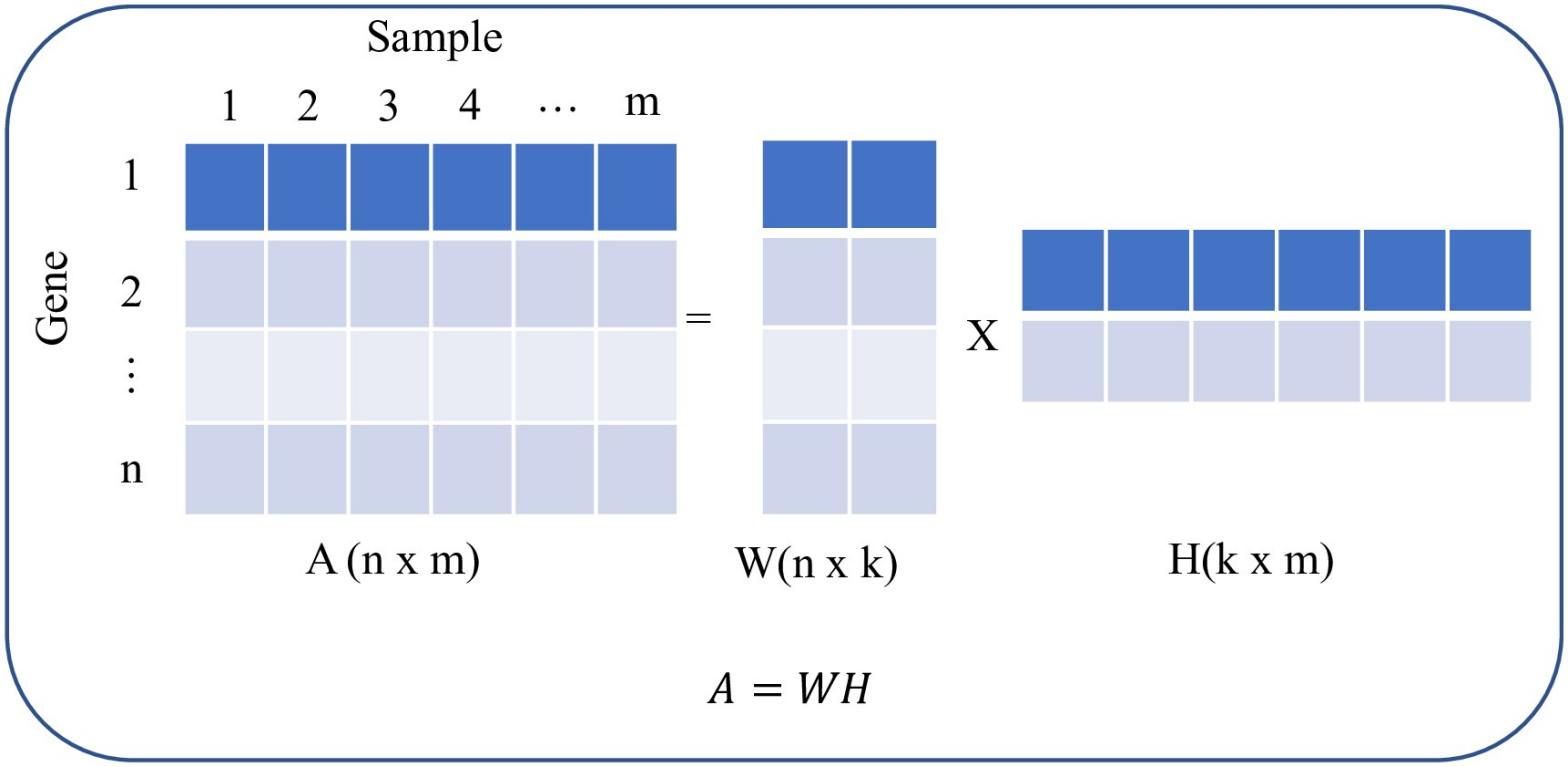
Non-negative matrix factorization procedure.

Among different variants of NMF, we employed divergence-based algorithm proposed by Lee and Seung [31] due to its simplicity and robustness [26, 31]. Briefly, given a gene expression matrix A of size *n* × *m* (*n* genes and *m* samples) and desired number of clusters *k*, the NMF decomposes A into two non-negative matrices W (*n* × *k*) and H (*k* × *m*) (Fig 2).

W and H matrix are computed using iterative method to minimize the following cost function.

$$D=\sum_{ij}{(A}_{ij}log\frac{A_{ij}}{{\left(WH\right)}_{ij}}-A_{ij}+\;{WH}_{ij})$$

In each iteration, W and H are updated using following multiplicative updating rules,

$$H_{au}\;\leftarrow \;H_{au}\;\frac{\sum_{i}W_{ia}A_{iu}/{\left(WH\right)}_{iu}}{\sum_{k}W_{ka}}$$

$$W_{ia}\;\leftarrow \;W_{ia}\;\frac{\sum_{u}H_{au}A_{iu}/{\left(WH\right)}_{iu}}{\sum_{v}W_{av}}$$

Cluster membership of each sample is assigned based on the row index of maximal number in the column of H matrix.

#### Consensus-matrix based model selection

We used consensus matrix-base model selection strategy to select best number of clusters [26]. For a given number of clusters K, NMF groups the samples into K clusters. A total of 40 NMF runs were employed to construct the consensus matrix *C* (*n* × *n*). Each element of consensus matrix represents the probability that two samples cluster together. Then, the cophenetic correlation coefficient ρ_k_ was computed as the Pearson correlation of the distance matrix between samples induced by the consensus matrix, i.e., *I* − *C*, and the distance matrix induced by the hierarchical clustering of *I* − *C*. ρ_k_ measures how faithfully a dendrogram preserves the pairwise distances in the consensus matrix and was calculated using the cophenet function in the scikit-learn library [32]. The best clustering is based on the value of ρ_k_.

### Identification of molecular subtype-specific signatures

To identify molecular subtype-specific signatures, we first computed the silhouette for each sample using following equation.

$$s(i)=\frac{b(i)-a(i)}{max\{a(i),\;b(i)\}}$$

Where *a*(*i*) and *b*(*i*) were computed as following,

$$a(i)=\frac{1}{|C_{i}|-1}\sum_{j∋C_{i},i\neq j}d(i,j)$$

$$b(i)=\underset{k\neq i}{\text{min}}\frac{1}{|C_{k}|\;}\sum_{j∋C_{k}}d(i,\;j)$$

*a*(*i*) is the mean distance of a sample to all other samples in the same cluster. It measures how well a sample is assigned to its own cluster. The smaller the value is, the better the assignment is. *b*(*i*) is the smallest mean distance of a sample to all samples in any other cluster. |*C*_*i*_| is the number of samples in its own cluster, |*C*_*k*_| is the number of samples in any other cluster, and *d*(*i*, *j*) is the distance of two samples computed with Euclidean distance.

The silhouette is a measure of how similar a sample is to its own cluster compared to other clusters. After removing outlier samples with negative silhouette width from each subtype, we applied statistical package edgeR (version: 3.28.0) to obtain pairwise differentially expressed genes (DEGs) between molecular subtypes. To facilitate downstream analysis of molecular subtypes, we used fold change of 1.5 and false discover rate (FDR) of 0.05 as cutoffs. We define the gene signature of each subtype as DEGs that have the highest value in each molecular subtype.

### Pathway enrichment analysis

A Bioconductor package clusterProfiler (Version 3.14.3) [33] was used to perform pathway enrichment analysis for each identified molecular subtype. ClusterProfile is a statistical package that integrates several ontologies, including Gene Ontology, Disease Ontology, and KEGG pathway, to perform over-representation analysis and gene set enrichment analysis.

### Validation of AD molecular subtype in independent datasets

Two independent datasets from Gene Expression Omnibus (GSE44770, GSE118553) were used for validation of AD molecular subtypes. GSE44770 includes gene expression data from frontal cortex of 230 subjects, 128 of which are late-onset Alzheimer´s disease (LOAD) patients. GSE118553 includes gene expression data from frontal cortex of 112 subjects, including 52 AD patients. We used normalized data from GSE44770 and GSE118553 to validate molecular subtypes identified based on ROSMAP data.

Since ground truth of clusters in a dataset is unknown, there are no quantitative method to formally validate clusters in an independent dataset. Therefore, visualization is suggested as a valid approach [34]. A discovery by signature gene strategy proposed by other studies was used for this validation [27, 28]. The basic idea of this strategy is that using the signature gene from the discovery dataset to cluster a new dataset to see if the signature gene expression shows similar patterns with the discovery dataset. It includes three steps. First, signature genes were projected onto normalized independent dataset and consensus NMF clustering was used to identify number of clusters. Second, molecular subtype identity was assigned using signature genes. Third, a heatmap of signature gene expression was then generated to visualize the molecular subtype. In addition, we performed pathway enrichment analysis to further confirm the molecular subtypes in independent datasets.

### Correlation of AD molecular subtype with patient demographics, clinicopathology, and APOE genotype

We examined the demographic distributions of AD molecular subtype, including age, sex, race and education, and assessed the associations of AD molecular subtype with APOE genotype and clinical variables, including Braak stage, The Consortium to Establish a Registry for Alzheimer’s Disease (CERAD) diagnosis, and Mini-Mental State Examination (MMSE) score. The Braak stage is a semiquantitative measure of severity of neurofibrillary tangle (NFT) pathology [35, 36]. Braak stages I and II indicate NFTs confined mainly to the entorhinal region of the brain. Braak stages III and IV indicate involvement of limbic regions such as the hippocampus. Braak stages V and VI indicate moderate to severe neocortical involvement. CERAD score is a semiquantitative measure of neurotic plaques [37]. Based on semiquantitative estimates of neurotic plaque density, a neuropathologic diagnosis was made of no AD, possible AD, probable AD, or definite AD. MMSE test is a 30-point questionnaire that is used extensively in clinical and research settings to measure cognitive impairment.

For categorical variables, including Braak stage, CERAD, and APOE, Fisher’s exact test was used to assess their associations with AD molecular subtype. For continuous variables, such as MMSE and education, student’s t-test was used. All statistical analysis was performed using R (version: 3.6.2). Significance level was defined as p value less than 0.05.

### Ethics statement

This is a secondary research use for ROSMAP data and patient information is not identifiable. The IRB at Case Western Reserve University determined that the proposed activity is not research involving human subjects and IRB review and approval is not required (STUDY20190935). Therefore, patient consent is not applicable or not required.

## Results

### AD consists of two molecular subtypes

**Fig 3:**
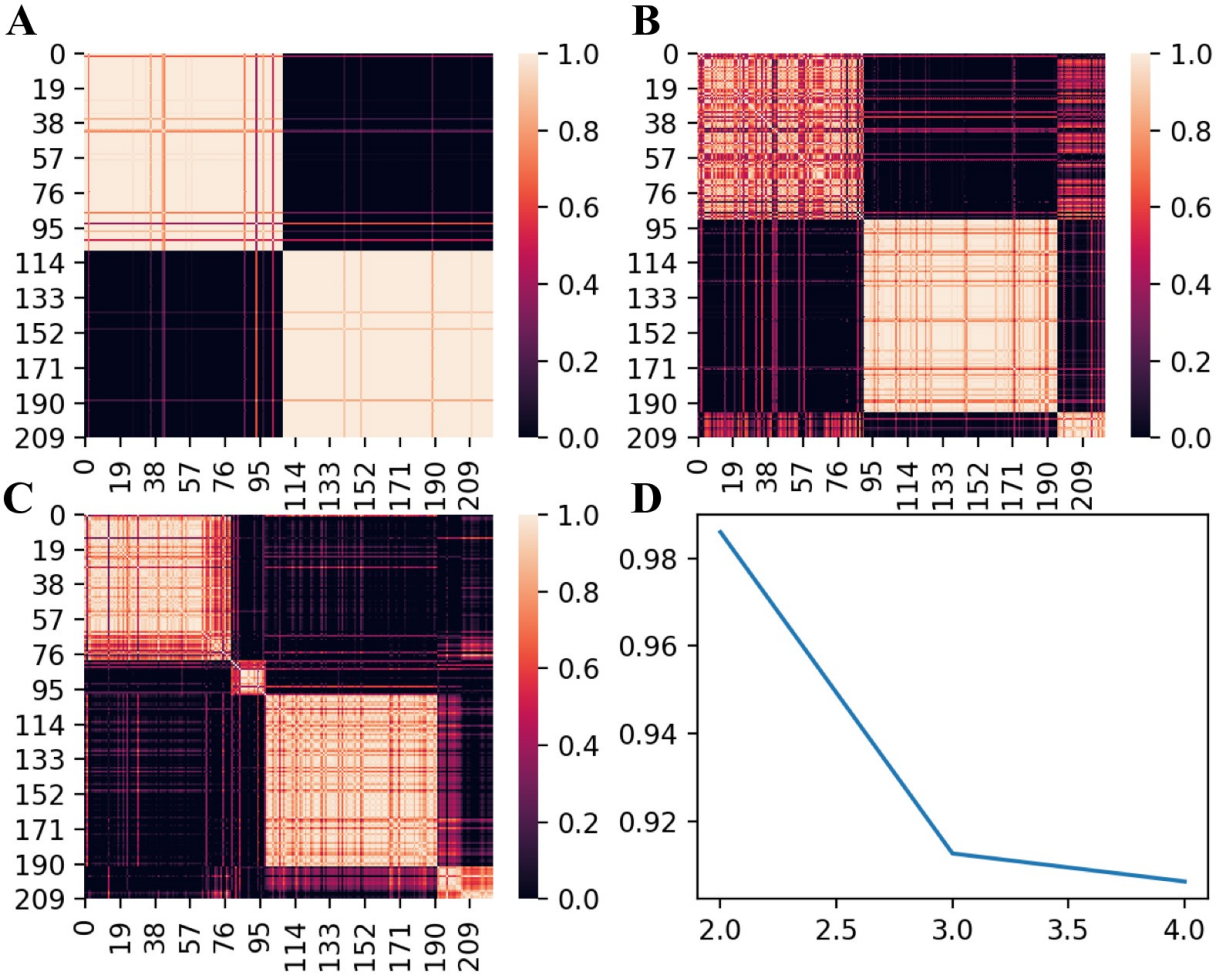
NMF-based clustering of gene expression data from 222 AD patients in ROSMAP. (A-C) Consensus matrices for 2, 3 and 4 clusters respectively. (D) Plot of cophenetic correlation coefficient against the number of clusters.

**Fig 4:**
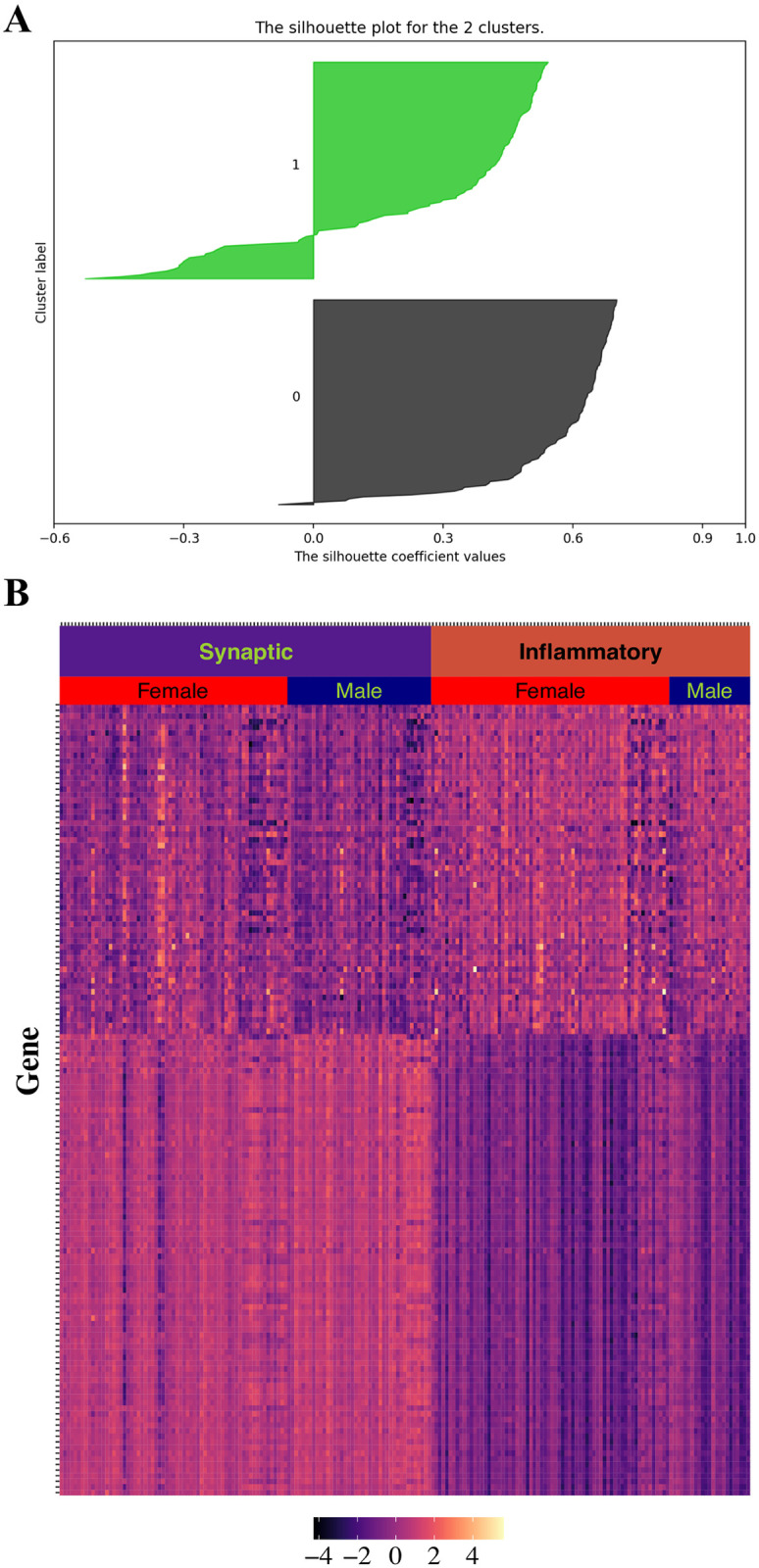
Signature genes in each molecular subtype. (A) Silhouette score for each sample (B) Heatmap for signature gene expression in each molecular subtype. Gene expression is represented as normalized value.

We used consensus NMF to cluster gene expression data of 222 AD patients from ROSMAP. Compared with three and four clusters, consensus matrix from two clusters are more stable (Fig 3A–3C). In addition, cophenetic correlation coefficient drops when we assign the data into three subtypes (Fig 3D). These evidences indicate that patient data can be best represented by two distinct subtypes. We obtained 403 differentially expressed genes between these two molecular subtypes as signature genes using 197 core samples with positive silhouette score (Fig 4A). We can see the distinct pattern of signature gene expression in these two subtypes (Fig 4B).

We named the molecular subtypes according to signature genes up-regulated in each cluster. For synaptic type, highly expressed genes are associated with synapse function, such as SNAP25, RAB3A, VAMP1, SYNJ1, and STXBP1. A total of 37 pathways were significantly enriched and 23 of 37 (62.2%) are related to synapse function (S1 Table). The top 10 enriched pathways of this subtype are shown in Table 1. We can see that synaptic type is characterized by dysfunction of synapse, including synaptic vesicle priming and recycling, and neurotransmitter secretion (Table 1).

**Table 1:** Top 10 enriched pathways in the synaptic type of AD.

*PATHWAY*	*P value (adjusted)*	*Fold enriched*
*Synaptic vesicle cycle*	4.1E-04	5.63
*Vesicle-mediated transport in synapse*	4.1E-04	5.36
*Synaptic vesicle priming*	1.4E-03	21.32
*Synaptic vesicle recycling*	1.4E-03	8.98
*Calcium ion regulated exocytosis*	1.4E-03	5.81
*Synaptic vesicle endocytosis*	3.0E-03	9.33
*Presynaptic endocytosis*	3.0E-03	9.33
*Neurotransmitter secretion*	3.3E-03	5.03
*Signal release from synapse*	3.2E-03	5.03
*Signal release*	1.1E-02	2.92

For inflammatory type, highly expressed genes are related to inflammatory pathways, such as BST2, GBP4, IFI44L, IFITM2, IFITM3, IL4R, IRF, MT2A, PSMB9, and TXNIP. A total of 3 pathways were significantly enriched using the signature genes. This subtype is characterized with dysfunction of inflammatory responses, including interferon alpha (IFN-α), interferon gamma (INF-γ) and IL2 pathways (Table 2).

**Table 2:** Enriched pathways in the inflammatory type of AD.

*PATHWAY*	*P value (adjusted)*	*Fold enriched*
*Interferon alpha response*	4.3E-05	7.83
*Interferon gamma response*	1.4E-03	4.22
*IL2-Stat5 signaling*	2.1E-02	3.37

### AD molecular subtypes were validated in independent datasets

**Fig 5:**
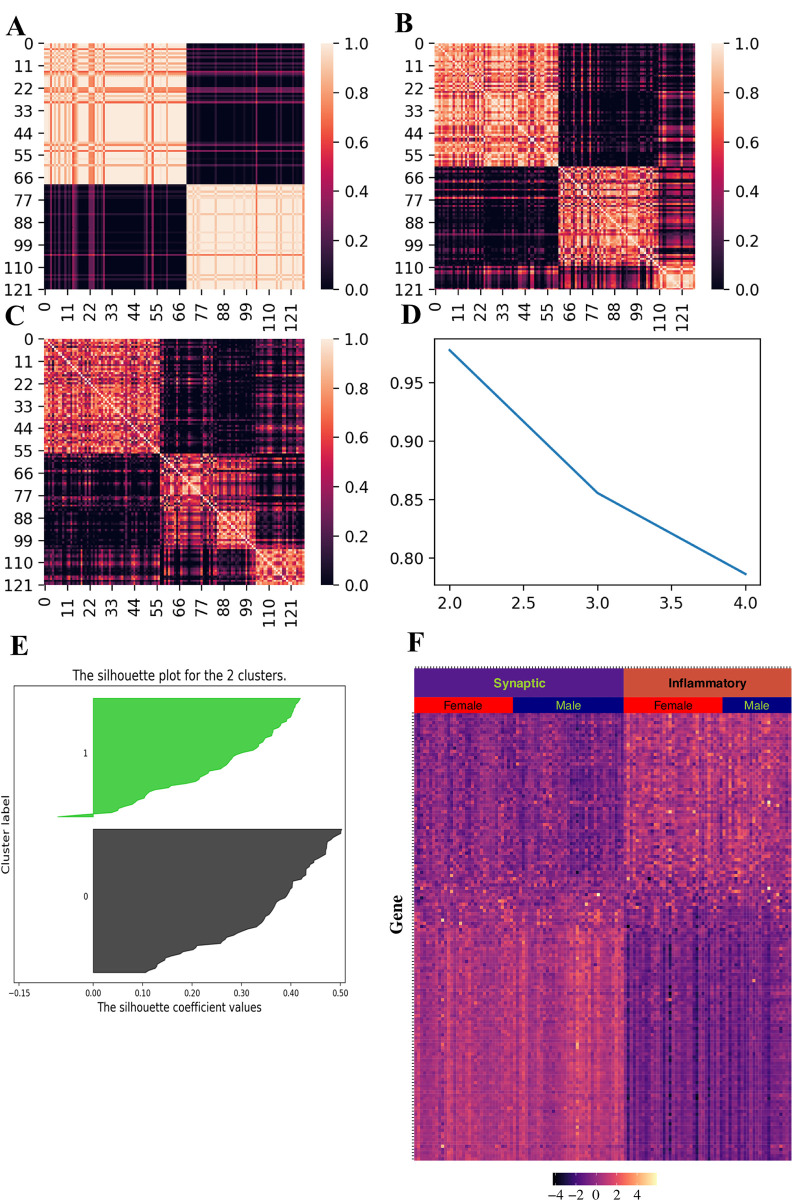
Molecular subtype validation in GEO dataset (GSE44770). (A-C) Consensus matrices for 2, 3 and 4 clusters respectively. (D) Plot of cophenetic correlation coefficient against the number of clusters. (E) Silhouette distance for each sample. (F) Heatmap for signature gene expression.

**Fig 6:**
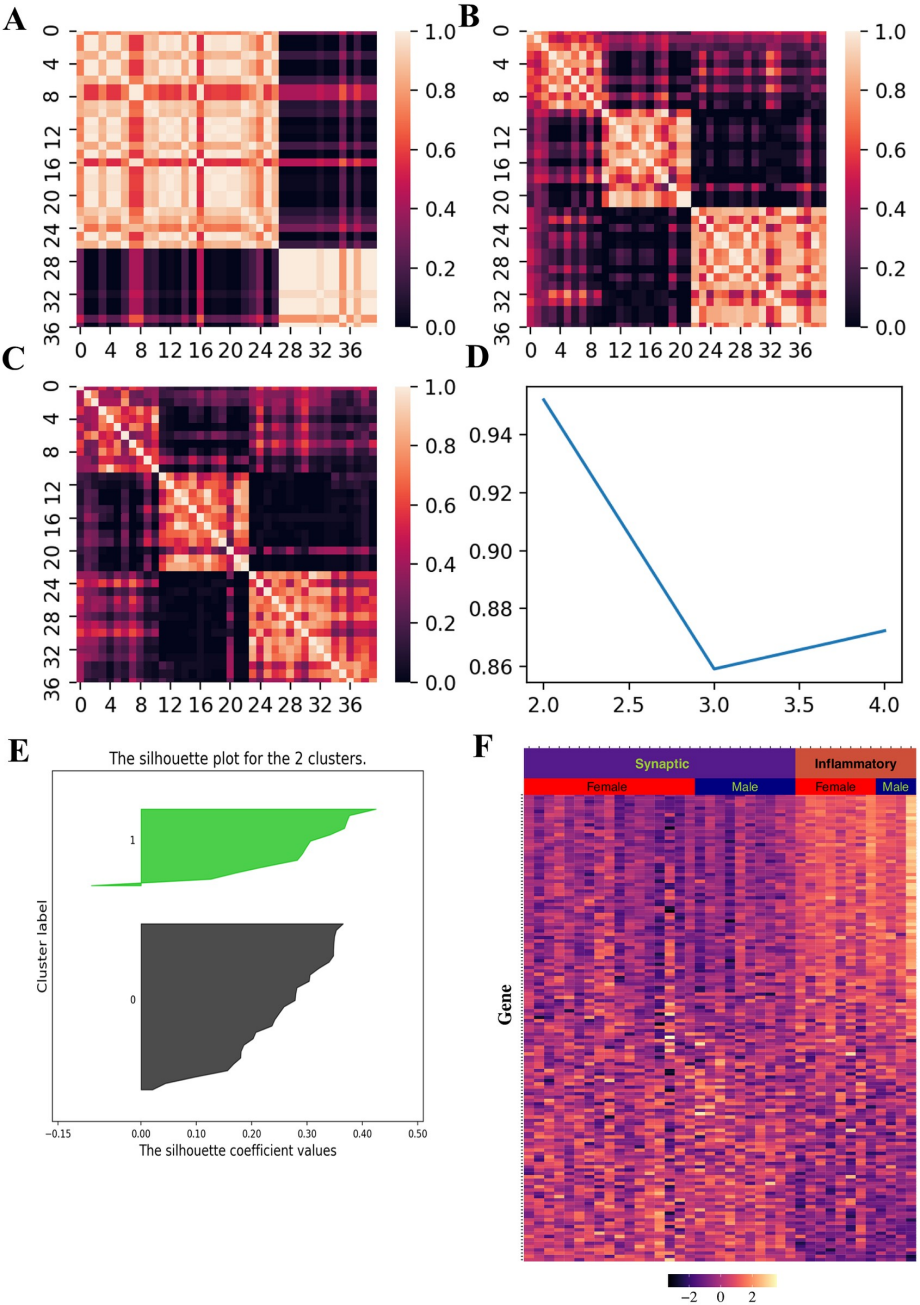
Molecular subtype validation in GEO dataset (GSE118553). (A-C) Consensus matrices for 2, 3 and 4 clusters respectively. (D) Plot of cophenetic correlation coefficient against the number of clusters. (E) Silhouette distance for each sample. (F) Heatmap for signature gene expression.

We validated the two AD molecular subtypes using two independent datasets from GEO (GSE44770, n = 128 and GSE118553, n = 40). Using consensus NMF, we identified clusters based on these two independent datasets from GEO (Figs 5 and 6). Majority of samples have positive silhouette scores (Figs 5E and 6E), indicating that samples are well classified using signature genes we obtained from ROSMAP. We can see distinct patterns for signature gene expression in these two clusters, indicating that these two clusters represent the same molecular subtypes from ROSMAP (Figs 5E and 6F).

To further validate the AD molecular subtypes in these two datasets, we performed pathway enrichment for each cluster in each dataset. For GSE44770 dataset, a total of 30 pathways were significantly enriched in first cluster (S2 Table). Seven of them exactly occur in enriched pathways of ROSMAP-based synaptic type AD and ten additional pathways are related to synaptic function, indicating that this cluster is a synaptic type. Ten pathways were enriched in second cluster and all three enriched pathways in ROSMAP-based inflammatory AD occur in this cluster, indicating that this cluster is an inflammatory type. Similar results were obtained in GSE118553 dataset. A total of ten pathways and two pathways were significantly enriched in each cluster respectively (S3 Table). In the first cluster, four of ten pathways are overlapped with the enriched pathways of ROSMAP-based synaptic type AD and five other pathways are related to synaptic function. In the second cluster, two enriched pathways are overlapped with the enriched pathways in ROSMAP-based inflammatory subtype.

### Association analyses of AD molecular subtype with patient demographics, clinicopathology, and APOE genotype

We investigated whether AD subtypes are associated with demographic and clinical variables using the core samples from ROSMAP dataset (197 patients). The distributions of AD molecular subtype in demographic variables, including age, race and education, show no significant difference (Table 3). Interestingly, we noticed that synaptic type AD is more prevalent than inflammatory type in male patients (p = 0.051). Several measurements for AD severity are available in ROSMAP, including AD Braak stage, CREAD score and MMSE score. We didn’t see significant associations of AD molecular subtype with these variables (Table 3). This result suggests that AD molecular subtype might be not related to AD severity, but caution should be taken when explaining this result due to small sample size. ROSMAP also includes APOE genotype, the most important genetic risk factor for late-onset AD. A significant association of AD molecular subtype with APOE was observed (p = 0.031). We can see that synaptic type AD is more prevalent in patients with E3E4 genotype and inflammatory type AD is more prevalent in patients with E3E3 genotype (Table 3).

**Table 3:** Association of AD molecular subtype with demographic, clinical variables and APOE genotype in the ROSMAP dataset.

	Synaptic type (Num. of Patients)	Inflammatory type (Num. of Patients)	p ^a^
**Age**			
< 65	0	0	1.0
65–80	5	4	
> 80	101	87	
**Sex**			
Female	65	68	**0.051**
Male	41	23	
**Race**			
White	104	90	1.0
Black	2	1	
**Education**	16.70	16.21	0.98
**Braak stage**			
I	4	2	0.88
II	4	2	
III	20	17	
IV	35	29	
V	41	37	
VI	2	4	
**CREAD score**			
Definite	48	50	0.47
Probable	44	28	
Possible	5	5	
No AD	9	8	
**MMSE**	13.84	12.23	0.19
**APOE**			
E2E2	0	1	**0.031**
E2E3	12	9	
E2E4	5	2	
E3E3	46	55	
E3E4	43	22	
E4E4	0	2	

^a^ For categorical variables, including Braak stage, CREAD score and APOE, p value was computed using Fisher’s exact test. For continuous variables, including Education and MMSE, the p value was computed using student’s t-test.

We then examined whether these associations can also be observed in the two validation datasets. Although we didn’t see a significant association of sex with molecular subtype, we observed that the synaptic type is more prevalent in male patients than in females in both datasets. In the GSE44770, 37 of 60 (61.7%) are synaptic type in male patients, while it is 33 of 66 (50.0%) in female patients. In the GSE118553, the prevalence of synaptic type in male and female patients are 10 of 14 (71.4%) and 17 of 25 (68.0%) respectively. Due to the lack of APOE genotype in these two datasets, we were unable to investigate the association of APOE with molecular subtype (Table 4).

**Table 4:** Association of AD molecular subtype with age and sex in the two validation datasets.

		Synaptic type (Num. of Patients)	Inflammatory type (Num. of Patients)	p
**GSE44770**	**Age**			
	< 65	6	4	0.963
	65–80	30	23	
	> 80	34	29	
	**Sex**			
	Female	33	33	0.212
	Male	37	23	
**GSE118553**	**Age**			
	< 65	0	1	0.495
	65–80	9	4	
	> 80	18	7	
	**Sex**			
	Female	17	8	1
	Male	10	4	

## Discussion

In this study, we applied non-negative matrix factorization combined with consensus matrix-based cluster selection and identified two molecular subtypes based on gene expression data of AD. Synaptic type is characterized by dysfunction of synaptic pathways. Substantial loss of neurons and synapses is a hallmark in late stage AD. Recent studies also show synaptic dysfunction was observed in mild cognitive impairment patients [38–40], suggesting that synaptic dysfunction is a fundamental mechanism of AD. On the other hand, inflammatory type is enriched with over-activation of IL-2, IFN-α, and IFN-γ pathways. The central role of inflammation in AD development is recently established [41–43]. A sustained inflammatory response, mediated by over-activation of microglia and other immune cells, has been demonstrated to exacerbate both amyloid and tau pathology [42]. Roy ER et al reported that IFN-α response drives neuroinflammation and grossly upregulated in AD [44]. A recent study links IL-2 pathway to amyloid pathology of AD [45]. All these evidences demonstrated that inflammation represents another mechanism of AD. Therefore, the two AD molecular subtypes we identified reflect inherent molecular mechanism of AD. Interestingly, two studies reported that microglia are involved in synaptic pruning and plays a role in pathological remodeling of neuronal circuits [46, 47], indicating that two molecular processes may be related.

GWAS has identified more than 40 genes/loci as the genetic risk factors of AD, which greatly expands our mechanistic understanding of the etiology of AD. While some of these genes/loci have been mapped to Aβ pathology, including amyloid precursor protein (APP) metabolism, Aβ aggregation, clearance, toxicity, and Tau pathology, a large amount of these genes is related to non-Aβ and -Tau pathways [48]. Lambert et al suggested that a common mechanism, i.e., focal adhesion pathway, may link Aβ and tau pathology and ultimately lead to synapse dysfunction. A shift from Aβ-centered hypothesis to synapse-centered hypothesis has emerged [48, 49]. Here, we used gene expression data to define two molecular subtypes of AD and enriched pathways high-lighten synapse dysfunction, which supports this synapse-centered hypothesis. Furthermore, our study implies two mechanisms for synaptic dysfunction. One is the aberrant synaptic pathways themselves, such as synaptic vesicle endocytosis and exocytosis. Another is the indirect mechanism through immune system dysfunction, which may affect Aβ clearance and synaptic pruning.

Using available patient clinical information, we evaluated their associations with molecular subtypes. We didn’t find significant correlation of molecular subtype with severity of cognitive impairment. However, we were unable to control potential confounders due to very limited information available in the dataset. We show that AD molecular subtype is significantly associated with APOE genotype. APOE has three alleles, including E2, E3 and E4. APOE4 is the main genetic determinant for late-onset AD and individual with APOE4 significantly increases the risk of AD [50, 51]. While some studies show APOE4 promotes AD by interaction with Aβ, especially it hinders Aβ clearance [52], other studies link APOE4 with synaptic function, such as synapse recycling [53]. In this study, we observed that synaptic type of AD is more common in patients with E3E4 genotype. Although APOE is not in the list of signature genes, it may regulate synaptic function by interacting with downstream molecules including APOE receptor in the brain. This observation further supports synaptic mechanism of APOE4 in AD development.

We observed that inflammatory type of AD is more prevalent in women. On the other hand, synaptic type of AD is more prevalent in men. Sex differences in both synaptic plasticity and inflammatory response have been observed [54, 55]. Females often have strong both innate and adaptive immune responses [55]. This results in faster clearance of pathogens in females than males, but also contributes to increased susceptibility to inflammatory diseases in females, such as systemic lupus erythematosus and multiple sclerosis [56]. Since inflammation plays a central role in AD development, females are more likely to develop inflammatory type AD than males. Sex difference in dendrite spine density (DSD) in the hippocampus has been observed in animal models decades ago, which is regulated by steroid hormones and environmental stress. The female rats have double of DSD than males and DSD experienced dramatic changes during the estrous cycle [57, 58]. This structural change in the hippocampus was also observed in human women during the menstrual cycle [59]. Many animal studies showed that increased spine density is associated with memory enhancement [60]. Compared to females, males have lower DSD in the hippocampus. Besides, no periodic fluctuation of hormone in males may lead to less synapse plasticity of hippocampal neurons due to lack of “practicing”. We hypothesize that lower DSD and possibly less synapse plasticity may make males more vulnerable to hippocampus damage, which may explain why synaptic type AD is more common in males.

Identification of AD molecular subtype has an implication for better design in clinical trials. Currently, clinical trials for AD are based on different cognitive groups from mild, moderate, and severe AD. However, most of this symptom-based clinical trials for AD fails, reflecting a lack of mechanistic understanding of AD. A recent clinical trial about a monoclonal antibody solanezumab failed the phase III trials for mild to moderate AD [61], but later it was found that it has benefits for a subgroup of patients with mild symptoms [62], supporting that patient subgrouping is important. Molecular subtyping of AD patients provides an attracting strategy for patient stratification in clinical trials. We prospect that including molecular subtype in clinical trial may contribute to discover personalized treatments for AD.

One limitation of this study is that molecular subtyping is based on gene expression data from post-mortem brain tissue, which limits its clinical usage. Nevertheless, identified molecular subtypes will help to understand the mechanism of AD. In the future, developing a practical molecular subtyping system for AD is demanded. Proteomic data from cerebrospinal fluid and genotype data from blood could be useful for such purpose.

## Conclusions

In this study, we reported the first gene expression-based molecular subtyping of AD. Using consensus NMF, we identified two robust molecular subtypes-synaptic type and inflammatory type-that represent two fundamental mechanisms of AD. These molecular subtypes are associated with APOE genotype and exhibit sex difference in distribution. Identification of molecular subtypes may have an implication in better clinical trial design and personalized medicine for AD.

## Supporting information

S1 Table Pathways enriched in each cluster from ROSMAP. (XLSX)

S2 Table Pathways enriched in in each cluster from GSE44770. (XLSX)

S3 Table Pathways enriched in in each cluster from GSE118553. (XLSX)
